# Supplementary material for: Genetic determinants of pOXA-48 plasmid maintenance and propagation in Escherichia coli
Source: Nat Commun. 2025 Aug 19;16:7734. doi: 10.1038/s41467-025-62404-7 (PMC12365149; doi:10.1038/s41467-025-62404-7)
Supplement: Supplementary file 1 — Supplementary Information [file 41467_2025_62404_MOESM1_ESM.pdf]

Supporting information for

Genetic Determinants of pOXA-48 Plasmid Maintenance and Propagation in *Escherichia coli*

Yannick Baffert[1], Nathan Fraikin[1], Yasmine Makhloufi[1], Julie Baltenneck[2], Marie-Eve Val[3], Annick Dedieu-Berne[1], Jonathan Degosserie[4], Bogdan I. Iorga[5], Pierre Bogaerts[6], Erwan Gueguen[2], Christian Lesterlin[1] and Sarah Bigot[1*]

Affiliations:
[1] Microbiologie Moléculaire et Biochimie Structurale (MMSB), Université Lyon 1, CNRS, Inserm, UMR5086, 69007, Lyon, France
[2] Microbiologie Adaptation et Pathogénie (MAP), Université Lyon 1, INSA de Lyon, CNRS, UMR 5240, F-69622, Villeurbanne, France.
[3] Institut Pasteur, Université Paris Cité, CNRS UMR3525, Unité Plasticité du Génome Bactérien, Département Génomes et Génétique, Paris, France.
[4] Namur Molecular Tech, UCLouvain, CHU UCL Namur, 5530 Yvoir, Belgium
[5] Université Paris-Saclay, CNRS UPR 2301, Institut de Chimie des Substances Naturelles, Gif-sur-Yvette, France
[6] National reference center for antimicrobial resistance in Gram negative, CHU UCL Namur, 5530, Yvoir Belgium

* corresponding author: sarah.bigot@cnrs.fr

This PDF file includes:

Figures S1 to S5
Table S1 to S4
SI References

Figure S1

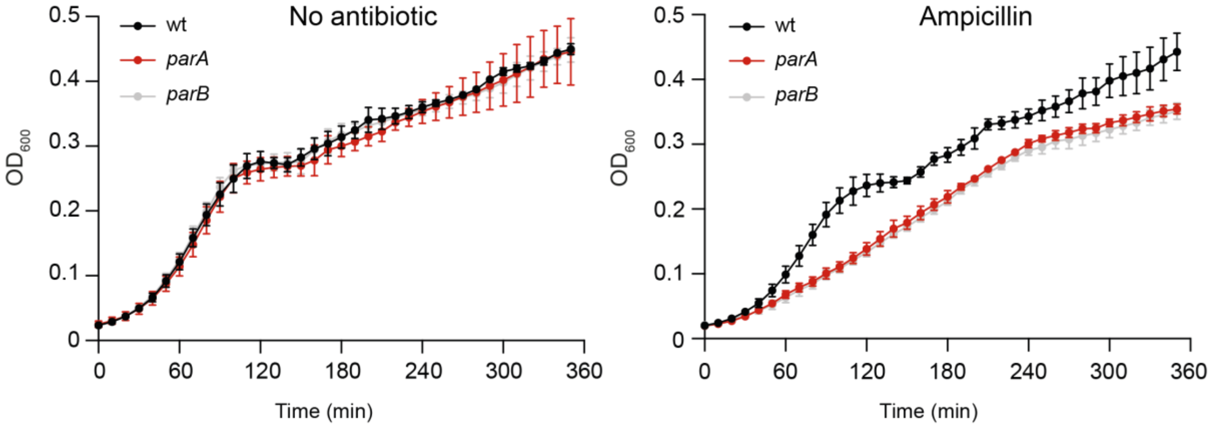

Figure S1. Impact of selective pressure on *parA* and *parB* mutant growth. Growth curve of wild-type (wt), *parA* and *parB* mutant strains represented as optical density (OD) at 600 nm wavelength measured during 360 min at 37°C in absence or presence of ampicillin. The mean and SD of three independent clones is represented for each strain.

Figure S2

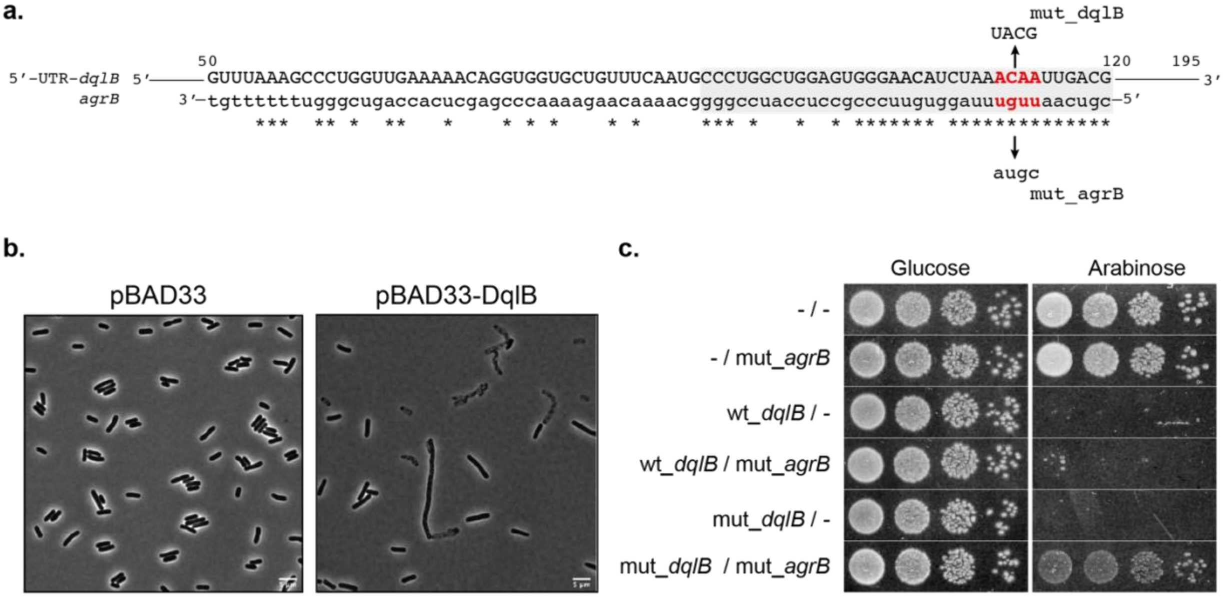

Figure S2. *agrB* RNA inhibits DqlB toxicity through specific base-pairing with the 5' UTR of the *dqlB* mRNA. a. Sequence alignment between the non-coding *agrB* RNA and the 5' UTR of the *dqlB* mRNA. The grey box highlights the 35-nt predicted base-pairing region. Identical nucleotides are marked with asterisk, and mutations introduced to disrupt pairing are shown in red. **b**. Effect of DqlB production on cell morphology. Representative phase contrast microscopy images of *E. coli* cells carrying either the empty vector pBAD33 (left; LY4076) or pBAD33-DqlB (right; LY4077), with the *dqlB* gene under the arabinose inducible PBAD promoter, after 4 h of induction with arabinose. Scale bar: 5 µm. **c**. Spot assays showing the importance of the sequence homology between the 5' UTR of *dqlB* mRNA and *agrB* RNA. Growth of *E. coli* (-/-;LY4078), *E. coli* producing mut_*agrB* (LY4424), wild-type wt_*dqlB* (LY4079), wt_*dqlB* and mut_*agrB* (LY4423), mut_*dqlB* (LY4545), or mut_*dqlB* and mut_*agrB* (LY4546) is shown under conditions with either glucose or arabinose.

Figure S3

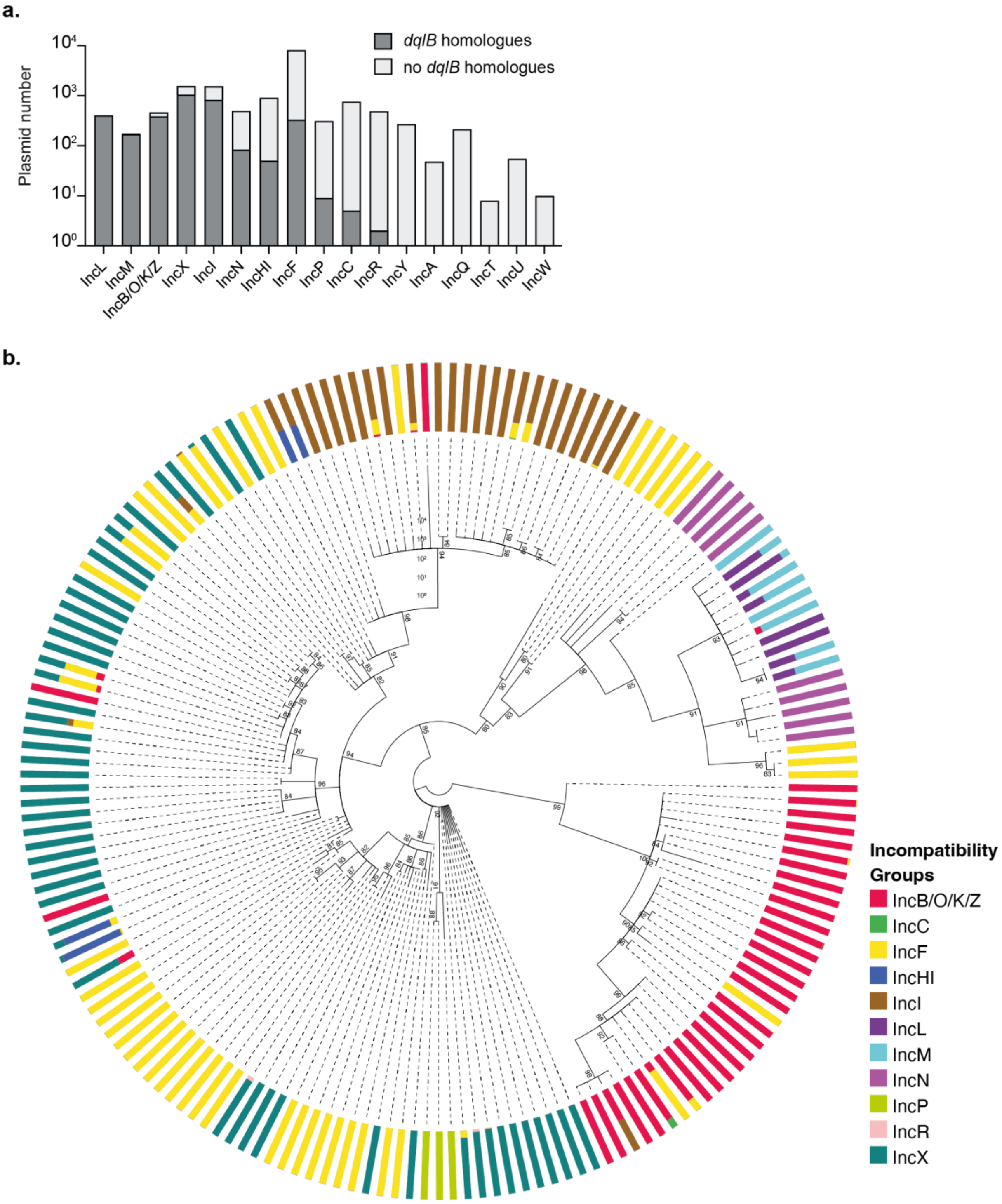

Figure S3. Phylogenetic analysis and distribution of *dqlB* homologues among conjugative plasmids. a. Distribution of *dqlB* homologues across different plasmid incompatibility groups. The number of plasmids within each incompatibility group is shown on the y-axis, with the presence (dark grey) or absence (light grey) of *dqlB* homologues indicated. **b**. Maximum likelihood phylogenetic tree of *dqlB* homologues identified from conjugative plasmid sequences. Branch support values were calculated using 1000 ultrafast bootstrap (UFboot). The outer ring color represents different incompatibility groups, demonstrating the phylogenetic relationships of *dqlB* homologues and their association with plasmid incompatibility groups.

Figure S4

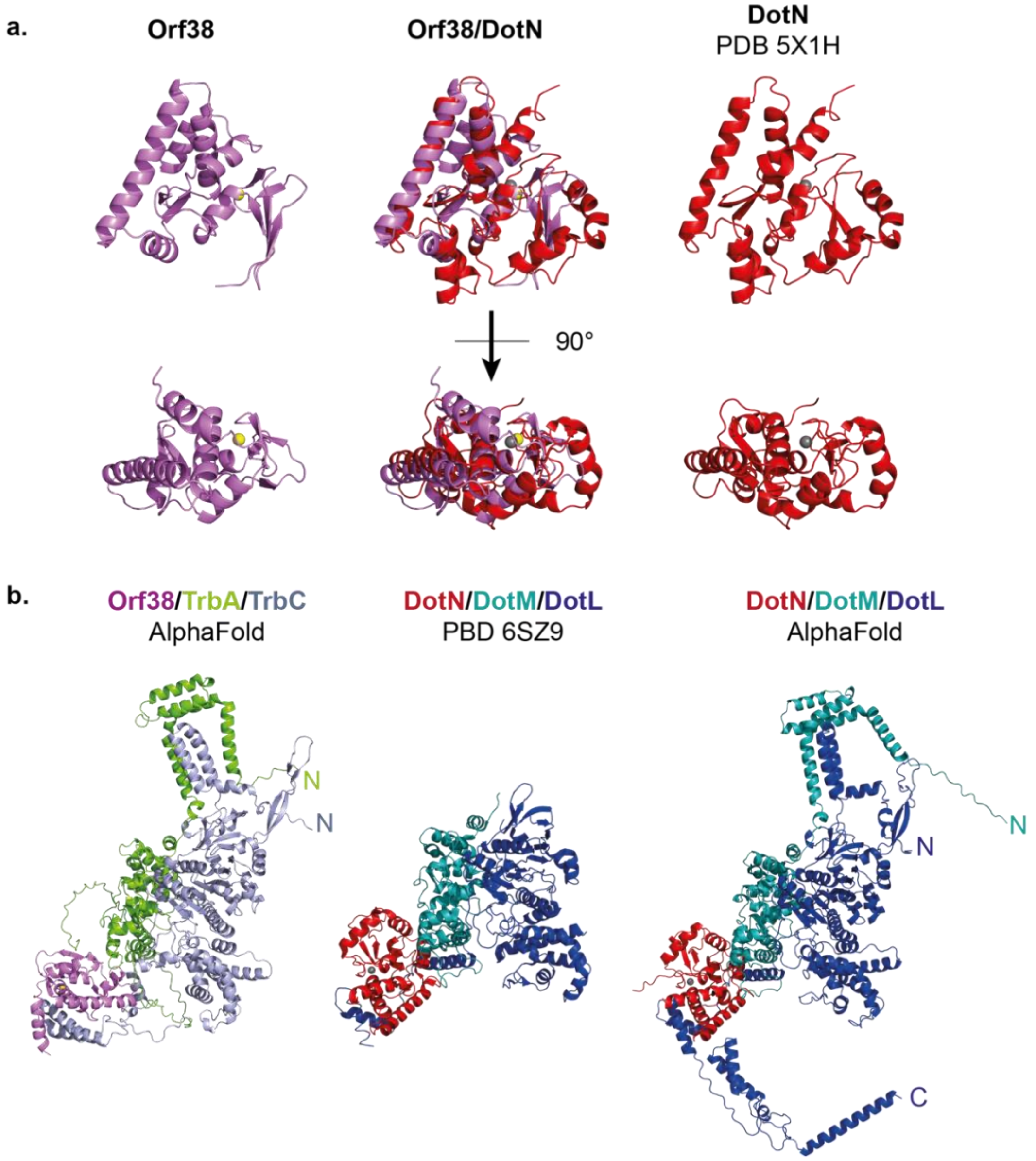

Figure S4. Comparison of the Orf38 and DotN of *Legionella pneumophila* structures. a. Left: predicted structure of Orf38 by AlphaFold 3.0; right: structure of DotN from *L. pneumophila* (PDB 5X1H); middle: structural alignment of the predicted Orf38 (purple) and DotN (red) using PyMoL. **b**. Comparison of the modeled Orf38/TrbA/TrbC complex with the DotN/DotM/DotL complex. Left: AlphaFold-predicted model of the Orf38/TrbA/TrbC complex, where Orf38 is shown in purple, TrbA in green, and TrbC in gray. Middle: Cryo-EM structure of the *L. pneumophila* DotN/DotM/DotL complex (PDB 6SZ9), with DotN in red, DotM in teal, and DotL in blue. Right: AlphaFold-predicted model of the DotN/DotM/DotL complex.

Figure S5

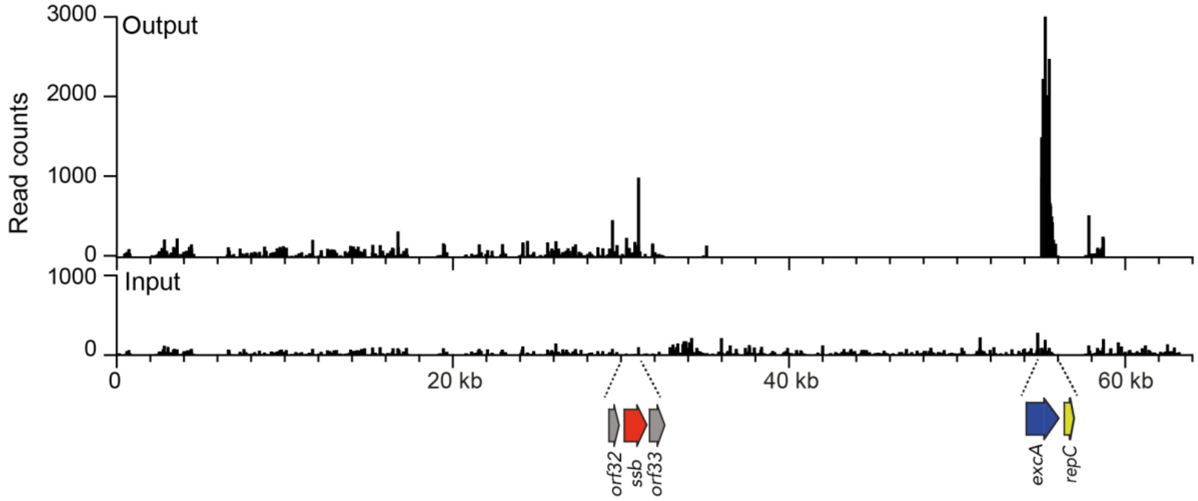

Figure S5. Enrichment of transposon insertions in the output library compared to the input library. Read distribution of transposon insertions across the pOXA-48 plasmid in both the input and output transposon libraries. The y-axis represents the number of reads, and the x-axis their position across the plasmid. Regions containing *orf32*, *ssb*, *orf33*, *excA*, and *repC*, with significant enrichment, are indicated.

Table S1. Predicted proteins encoded by the pOXA-48 plasmid

Protein	Conserved domain	Putative function	Vir / Dot T4SS homologue	% identity pCTX-M3
Orf67	-	-	-	-
Tir*	CPBP family	Fertility inhibition	-	93.5
PemI	MazE superfamily	Type II antitoxin	-	98.8
PemK	CcdB superfamily	Type II toxin	-	99
MucA	LexA superfamily	Transcriptional repressor LexA	-	98.6
MucB	PolY superfamily	Translesion synthesis	-	98.4
Orf5	-	-	-	100
Orf6	-	-	-	98.6
Orf7	-	-	-	98.7
Orf8	HTH-XRE superfamily	Gene regulation	-	94.4
Orf9	RelB Antitoxin	-	-	90.6
Orf10	-	-	-	89
Orf10.1	-	-	-	-
Orf11	-	-	-	90.2
Orf13	-	-	-	72.9
Orf14	-	-	-	96.7
Orf15	HTH-XRE superfamily	Gene regulation	-	98.8
Orf16	Restriction nuclease	Host defense	-	99
Orf17	-	-	-	93.2
Orf18	-	-	-	95.1
ResD	XerD superfamily	Dimer resolution	-	98.4
ParA	StbA/ParM like	Partition	-	99.7
ParB	Plasmid_stab_B superfamily	Partition	-	98.6
Nuc	PLDc_Nuc	Nuclease	-	96.2
Orf19	-	-	-	99.4
Orf20	YiaG- HTH-XRE superfamily	Gene regulation	-	99.4
Orf21	-	-	-	97.8
Orf22	-	-	-	98.8
Orf23	DUF308	-	-	99.2
Orf24	RadC superfamily	-	-	98.7
KorC	HTH-XRE superfamily	Gene regulation	-	100
Orf25*	DndB superfamily	-	-	100
CcgA1	-	-	-	91.5
Orf26	DUF4942	-	-	96.5
Orf27	-	-	-	98.2
RmoA	HHA superfamily	Gene regulation	-	98.7
Orf27.1	-	-	-	98.3
Orf28	-	-	-	96.1
Orf29	DUF1380	-	-	98.5
Orf30	-	-	-	99
KlcA	Antirestriction superfamily	Host defense	-	95.2
Orf31	-	-	-	98.9
Orf32	-	-	-	98.6
Ssb	RPA_2b-aaRSs_OBF_like superfamily	Single strand binding protein	-	97.9
Orf33	-	-	-	99
Orf34	-	-	-	99.4
Orf35	-	-	-	97.5
NikA	MobA superfamily	Reloxosome complex	-	100
NikB	PRK13878 superfamily	Relaxase	VirD2	99.7
TraH	DotD superfamily	Outer membrane complex	DotD	100
TraI	TraI superfamily	Outer membrane lipoprotein	VirB7 / DotC	100
TraJ	Plasmid_TraJ superfamily	ATPase	VirB11 / DotB	100
TraK	IcmT/TraK	ATPase	IcmT	100
Pri	COG4643/LPD7 superfamily	Primase	-	91.2
TraL	-	-	-	100
Orf36.1	Hha superfamily	Gene regulation	-	100
Orf36.2	HNS_MvaT superfamily	Gene regulation	-	100
TraM	IcmL/DotI superfamily	Inner membrane complex	VirB8 / DotI	99.6
TraN	IcmK/DotH superfamily	Outer membrane complex	VirB9 / DotH	99.7
TraO	TraO_IncI1 superfamily	Cell envelope-spanning channel	VirB10 / DotG	98.4

TraP	TraP_IncI1 superfamily	Pilus assembly	VirB3 / DotF	94.9
TraQ	TraQ_IncI1 superfamily	-	-	99.4
TraR	DUF6750	-	-	93
Orf38	-	-	-	99.4
TraU	VirB4 superfamily	ATPase	VirB4 / DotO	99.2
TraW	TraW superfamily	-	-	100
TraX	-	-	-	53.7
TraY	DotA/TraY superfamily		DotA	78.7
ExcA	-	Entry exclusion	-	34
RepC	RepC_IncL/M superfamily	Plasmid copy number	-	100
RepB	Leader_RepB_LM superfamily	Replication	-	87.5
RepA	IncFII_repA superfamily	Replication	-	99.4
TrbC	TrbC superfamily	Type IV Coupling protein	VirD4 / DotL	99.4
TrbB	Thioredoxin_like superfamily	-	-	98.7
TrbA	DotM superfamily	-	DotM	99.3
TrbN	Lys-like superfamily	Lytic transglycosylase	-	97.7

Elements of the Tn*1999* transposon and IS are not present. *gene encoding the protein is interrupted. Proteins that have been identified as being involved in plasmid maintenance and transfer are highlighted in yellow and blue, respectively.

Table S2. Strain list.

Name	Genotype	Source or construct
MS388	*E. coli* K-12 MG1655 *rpsL* (St^R)	Gift from F. Cornet
DY330	*W3110 ΔlacU169, gal490, λcI857, Δ(cro-bioA)*	[1]
DH5α	F⁻ *endA1 glnV44 thi-1 recA1 relA1 gyrA96 deoR nupG purB20* φ80d*lacZ*ΔM15 Δ(*lacZYA-argF*)U169, hsdR17($r_K^- m_K^+$), λ	Lab collection
LY1898	MFDpir / pSAM_Ec (Ap^R, Kan^R, DAP-)	[2]
LY945	MS388 *ilvA::Cm* (St^R, Cm^R)	[3]
LY1413	*E. coli* Top10 / pOXA-48 (Ap^R)	[4]
LY1507	MS388 *ilvA::erm* / pOXA-48 (St^R, Erm^R, Ap^R)	LY1413 x LY832 to Erm^R Ap^R
LY1672	DY330 / pOXA-48 (Ap^R)	LY1507 x DY330 to Ap^R lac-
LY1844	MS388 / pOXA-48 (St^R, Ap^R)	LY1672 x MS388 to Ap^R St^R
LY3154	DY330 / pOXA-48-*ΔparA::frt-kan-frt* (Ap^R, Kan^R)	λRed *frt-kan-frt* (OL1616/OL1617) at the endogenous pOXA-48 locus in LY1672
LY3162	MS388 / pOXA-48-*ΔparA::frt-kan-frt* (St^R, Ap^R, Kan^R)	Conjugation LY3154 x MS388 to St^R Kan^R
LY3257	MS388 / pOXA-48-*ΔparA::frt* (St^R, Ap^R)	Derivative of LY3162, *kan* removed via pCP20
LY3155	DY330 / pOXA-48-*ΔparB::frt-kan-frt* (Ap^R, Kan^R)	λRed *frt-kan-frt* (OL1620/OL1621) at the endogenous pOXA-48 locus in LY1672
LY3163	MS388 / pOXA-48-*ΔparB::frt-kan-frt* (St^R, Ap^R, Kan^R)	Conjugation LY3155 x MS388 to St^R Kan^R
LY3352	MS388 / pOXA-48-*ΔparB::frt* (St^R, Ap^R)	Derivative of LY3163, *kan* removed via pCP20
LY3156	DY330 / pOXA-48-*Δnuc::frt-kan-frt* (Ap^R, Kan^R)	λRed frt-kan-frt (OL1622/OL1623) at the endogenous pOXA-48 locus in LY1672
LY3164	MS388 / pOXA-48-*Δnuc::frt-kan-frt* (St^R, Ap^R, Kan^R)	Conjugation LY3156 x MS388 to St^R Kan^R
LY3258	MS388 / pOXA-48-*Δnuc::frt* (St^R, Ap^R)	Derivative of LY3164, *kan* removed via pCP20
LY4049	DY330 / pOXA-48-*Δorf19::frt-kan-frt* (Ap^R, Kan^R)	λRed *frt-kan-frt* (OL2218/OL2219) at the endogenous pOXA-48 locus in LY1672
LY832	MS388 *ilvA::erm* (St^R, Erm^R)	[5]
LY4141	MS388 *ilvA::erm* / pOXA-48-*Δorf19::frt-kan-frt* (St^R, Erm^R, Ap^R, Kan^R)	Conjugation LY4049 x LY832 to Erm^{R}Kn^R
LY2172	DY330 / pOXA-48-P$_{Tet}$-*sfgfp*-frt-kn-frt (Ap^R, Kan^R)	λRed *frt-kan-frt* (OL974/OL975) at the *orf27*.1 pOXA-48 locus in LY1672
LY4146	DY330 / pOXA-48-P$_{Tet}$-*sfgfp*-frt-kn-frt-*Δorf20::frt-cat-frt* (Ap^R, Kan^R, Cm^R)	λRed *frt-cat-frt* (OL2220/OL2221) at the endogenous locus in LY2172
LY681	MS388 *ilvA*::P$_{lacIQ1}$-tetR-frt-kan-frt (St^R)	[3]

LY699	MS388 *ilvA::*P_{lacIQ1}-tetR (St^R)	Derivative of LY681, *kan* removed via pCP20
LY4138	MS388 *ilvA::*P_{lacIQ1}-tetR / pOrf20 (St^R, Gm^R)	Transformation LY699 with pOrf20
LY4161	MS388 *ilvA::*P_{lacIQ1}-tetR / pOrf20 / pOXA-48-P_{Tet}-*sfgfp*-frt-kn-frt-*Δorf20::frt-kan-frt* (St^R, Ap^R, Kan^R, Cm^R, Gm^R)	Conjugation LY4146 x LY4138 to Gm^R Kan^R Cm^R
LY1593	MS388 *ilvA*::P_{tac}-mcherry (St^R, Gm^R)	[5]
LY4140	MS388 *ilvA*::P_{tac}-mcherry / pOrf20(St^R)	Transformation LY1593 with pOrf20
LY4129	DY330 / pOXA-48-*Δorf32::frt-kan-frt* (Ap^R, Kan^R)	λRed *frt-kan-frt* (OL2284/OL2285) at the endogenous pOXA-48 locus in LY1672
LY4132	MS388 / pOXA-48-*Δorf32::frt-kan-frt* (St^R, Ap^R, Kan^R)	Conjugation LY4129 x MS388 to St^R Kan^R
LY4148	MS388 / pOXA-48-*Δorf32::frt* (St^R, Ap^R)	Derivative of LY4132, *kan* removed via pCP20
LY4192	MS388 / pOXA-48-*Δorf32::frt* / pOrf32 (St^R, Ap^R, Gm^R)	Transformation LY4148 with pOrf32
LY4130	DY330 / pOXA-48-*Δssb::frt-kan-frt* (Ap^R, Kan^R)	λRed *frt-kan-frt* (OL2286/OL2287) at the endogenous pOXA-48 locus in LY1672
LY4133	MS388 / pOXA-48-*Δssb::frt-kan-frt* (St^R, Ap^R, Kan^R)	Conjugation LY4130 x MS388 to St^R Kan^R
LY4149	MS388 / pOXA-48-*Δssb::frt* (St^R, Ap^R)	Derivative of LY4133, *kan* removed via pCP20
LY4151	DY330 / pOXA-48-*Δorf33::frt-kan-frt* (Ap^R, Kan^R)	λRed *frt-kan-frt* (OL2288/OL2289) at the endogenous pOXA-48 locus in LY1672
LY4152	MS388 / pOXA-48-*Δorf33::frt-kan-frt* (St^R, Ap^R, Kan^R)	Conjugation LY4151 x MS388 to St^R Kan^R
LY4153	MS388 / pOXA-48-*Δorf33::frt* (St^R, Ap^R	Derivative of LY4152, *kan* removed via pCP20
LY3245	DY330 / pOXA-48-*Δorf36.1::frt-kan-frt* (Ap^R, Kan^R)	λRed *frt-kan-frt* (OL1721/OL1722) at the endogenous pOXA-48 locus in LY1672
LY3270	MS388 / pOXA-48-*Δorf36.1::frt-kan-frt* (St^R, Ap^R, Kan^R)	Conjugation LY33245 x MS388 to St^R Kan^R
LY3303	MS388 / pOXA-48-*Δorf36.1::frt* (St^R, Ap^R)	Derivative of LY3270, *kan* removed via pCP20
LY3697	MS388 / pOXA-48-*Δorf36.1::frt* /pOrf36.1 (St^R, Ap^R)	Transformation LY3303 with pOrf36.1c
LY3378	DY330 / pOXA-48-*Δorf38::frt-kan-frt* (Ap^R, Kan^R)	λRed *frt-kan-frt* (OL1825/OL1826) at the endogenous pOXA-48 locus in LY1672
LY3698	DY330 / pOXA-48-*Δorf38::frt-kan-frt* /pOrf38 (Ap^R, Kan^R, Gm^R)	Transformation LY3378 with pOrf38
LY3710	MS388 / pOXA-48-*Δorf38::frt-kan-frt* (St^R, Ap^R, Kan^R)	Conjugation LY3698 x MS388 to St^R Kan^R
LY3739	MS388 / pOXA-48-*Δorf38::frt* (St^R, Ap^R)	Derivative of LY3710, *kan* removed via pCP20
LY3747	MS388 / pOXA-48-*Δorf38::frt* /pOrf38(St^R, Ap^R)	Transformation LY3739 with pOrf38
LY3158	DY330 / pOXA-48-*ΔexcA::frt-kan-frt* (Ap^R, Kan^R)	λRed *frt-kan-frt* (OL1624/OL1625) at the endogenous pOXA-48 locus in LY1672
LY3166	MS388 / pOXA-48-*ΔexcA::frt-kan-frt* (St^R, Ap^R, Kan^R)	Conjugation LY3158 x MS388 to St^R Kan^R
LY3260	MS388 / pOXA-48-*ΔexcA::frt* (St^R, Ap^R)	Derivative of LY3166, *kan* removed via pCP20
LY3608	MS388 / pOXA-48-*ΔexcA::frt* / pExcA	Transformation LY3260 with pExcA
LY4164	Tn insertion *excA* clone 1	Tn-seq output library
LY4165	Tn insertion *excA* clone 2	Tn-seq output library
LY3157	DY330 / pOXA-48-*ΔrepC::frt-kan-frt* (Ap^R, Kan^R)	λRed *frt-kan-frt* (OL1626/OL1627) at the endogenous pOXA-48 locus in LY1672
LY3165	MS388 / pOXA-48-*ΔrepC::frt-kan-frt* (St^R, Ap^R, Kan^R)	Conjugation LY3157 x MS388 to St^R Kan^R
LY3259	MS388 / pOXA-48-*ΔrepC::frt* (St^R, Ap^R)	Derivative of LY3165, *kan* removed via pCP20
LY3606	MS388 / pOXA-48-*ΔrepC::frt* / pRepC	Transformation LY3259 with pRepC
LY1361	TB28 *ilvA::IsceICS- frt-cat-frt* / F-Tn*10* (Cm^{R,} Tc^R)	[5]
LY1369	TB28 *ilvA::IsceICS- frt-cat-frt* / F-Tn*10* / pBG29 (Cm^R, Tc^R, Kn^R)	[5]
LY1522	TB28 *ilvA::IsceICS- frt-cat-frt* / F-Tn*10* / pBG50 (Cm^R, Tc^R, Kn^R)	[5]

LY1549	TB28 *ilvA::IsceICS- frt-cat-frt* / F-Tn*10* / pBG52 (Cm^R, Tc^R, Kn^R)	[5]
LY4038	TB28 *ilvA::IsceICS- frt-cat-frt* / F-Tn*10* / TAP$_{kn}$-Cas9-OXA48-argB (Cm^R, Tc^R, Kn^R)	Transformation of LY1361 with TAP$_{kn}$-OXA48-*agrB*
LY4076	MS388 / pBAD33 (St^R, Cm^R)	Transformation MS388 with pBAD33
LY4077	MS388 / pBAD33-DqlB (St^R, Cm^R)	Transformation MS388 with pBAD33-DqlB
LY4078	MS388 / pBG50 / pBAD33 (St^R, Kn^{R,} Cm^R)	Transformation of MS388 with pBG50 and pBAD33
LY4079	MS388 / pBG50 / pBAD33-DqlB (St^R, Kn^{R,} Cm^R)	Transformation of MS388 with pBG50 and pBAD33-DqlB
LY4080	MS388 / TAP$_{kn}$-Cas9-OXA48-argB/ pBAD33 (St^R,Cm ^{R}, Kn^R)	Transformation of MS388 with TAP-OXA48-agrB and pBAD33
LY4081	MS388 / TAP$_{kn}$-Cas9-OXA48-argB / pBAD33-DqlB (St^R,Cm ^{R}, Kn^R)	Transformation of MS388 with TAP-OXA48-agrB and pBAD33-DqlB
LY4424	MS388 / TAP$_{kn}$-Cas9-OXA48-mut_argB/ pBAD33 (St^R,Cm ^{R}, Kn^R)	Transformation of LY4076 with TAP-OXA48-mut_agrB
LY4423	MS388 / TAP$_{kn}$-Cas9-OXA48-mut_argB / pBAD33-DqlB (St^R,Cm ^{R}, Kn^R)	Transformation of LY4077 with TAP-OXA48-mut_agrB
LY4545	MS388 / pBG50 / pBAD33-mut_dqlB (St^R, Kn^{R,} Cm^R)	Transformation of MS388 with pBG50 and pBAD33-mut_dqlB
LY4546	MS388 / TAP$_{kn}$-Cas9-OXA48-mut_argB / pBAD33-mut_dqlB (St^R,Cm ^{R}, Kn^R)	Transformation of MS388 with TAP$_{kn}$-Cas9-OXA48-mut_argB and pBAD33-mut_dqlB

Abbreviation *cat* and *kan* refers to insertion conferring resistance to chloramphenicol and kanamycin. St^R, Tc^R Ap^R, Cm^R, Kan^R and Erm^R refer to streptomycin, tetracycline, ampicillin, chloramphenicol, kanamycin and erythromycin resistances, respectively. *frt* refers to the FLP site-specific recombination site.

Table S3: plasmid list.

Name	Usage	Reference or construct
pOXA-48	Carries the *bla*$_{OXA-48}$ gene that encodes the OXA48 carbapenemase	[4]
F-Tn*10*	Template for P$_{Tet}$ promoter	[3]
pSAM_Ec	Carries the Himar-1 transposase used for Tn-Seq library, Ap^R, Kan^R	[6]
pKD3	Template plasmid for *frt*-flanked *cat* cassette, Cm^R	[7]
pR6K-sfGFP	Template plasmid for *frt*-flanked *kan* cassette, Kan^R	[3]
pCP20	Flp expression plasmid, Ap^R, Cm^R, ts	[7]
pUC18T-mini-Tn7T-Gm-Dsredexpress	Carries the *PA1/04/03* promoter, Ap^R, Gm^R	[8]
pUA66-psulA-gfp-Gm	Carries the pSC101 *oriV* and *acc3* gene, Gm^R	Derivative of pUA66[9]
pTrc99a	Carries the *lacI* gene, Ap^R	GE healthcare
pR6K-ptet-sfGFP	Carries the *sfgfp* gene under the P$_{Tet}$ promoter, Kan^R	Insertion of P$_{Tet}$ from F-Tn*10* upstream of *sfgfp* pR6K-sfGFP
pOrf20	Carries *orf20* gene under the *PA1/04/03* promoter, Gm^R	Insertion of *orf20* from pOXA-48 in pSC101-pPA
pOrf36.1 (pBG77)	Carries *orf36.1* gene under the *PA1/04/03* promoter, Gm^R	Insertion of *orf36.1* from pOXA-48 in pSC101-pPA
pOrf38 (pBG79)	Carries *orf38* gene under the *PA1/04/03* promoter, Gm^R	Insertion of *orf38* from pOXA-48 in pSC101-pPA
pExcA	Carries *excA* gene he *PA1/04/03* promoter, Gm^R	Insertion of *excA* from pOXA-48 in pSC101-pPA
pRepC	Carries *repC* gene under the *PA1/04/03* promoter, Gm^R	Insertion of *repC* from pOXA-48 in pSC101-pPA
pOrf32	Carries *orf32* gene under the *PA1/04/03* promoter, Gm^R	Insertion of *orf32* from pOXA-48 in pSC101-pPA
TAP$_{kn}$-Cas9-nsp (pBG29)	Produces Cas9 and nsp spacer, Kn^R	[5]

TAP_{kn}-Cas9-OXA48 (pBG50)	Produces Cas9 with OXA48 spacer targeting the promoter region of *bla*$_{OXA48}$, Kn^R	[5]
TAP_{kn}-Cas9-OXA48-pemI (pBG52)	Produces PemI and Cas9 with OXA48 spacer targeting the promoter region of *bla*$_{OXA48}$, Kn^R	[5]
TAP_{kn}-Cas9-OXA48-pemI-agrB	Produces PemI and *agrB* and Cas9 with OXA48 spacer targeting the promoter region of *bla*$_{OXA48}$, Kn^R.	Insertion *agrB* under its own promoter from pOXA-48 in pBG52
TAP_{kn}-Cas9-OXA48-agrB	Produces *agrB* and Cas9 with OXA48 spacer targeting the promoter region of *bla*$_{OXA48}$, Kn^R.	Deletion *pemI* in TAP_{kn}-Cas9-OXA48-pemI-agrB
TAP_{kn}-Cas9-OXA48-mut_argB	Produces *agrB* mutant, Kn^R.	Mutation 5'-ttgt-3' to 5'-cgta-3' in *argB* sequence
pBAD33-DqlB	Carries *dqlB* gene under the P_{BAD} promoter. Cm^R	Insertion *dqlB* from pOXA-48 in pBAD33 plasmid
pBAD33-mut_dqlB	Carries *dqlB* gene with the 5' UTR mutation. Cm^R	Mutation 5'-acaa-3' to 5'-tacg-3' in 5' UTR of *dqlB*

Abbreviations: *acc3*, *cat* and *kan* refer to gene conferring resistance to gentamycin, chloramphenicol and kanamycin respectively. Tc^R, Amp^R, Cm^R, Gm^R and Kan^R refer to tetracycline, ampicillin, chloramphenicol, gentamycin and kanamycin resistances respectively. *frt* and ts refer to the FLP site-specific recombination site and temperature sensitive.

Table S4: primer list.

Primer	Sequence	Construct
OL1306	ATGCAAGCTCTAGAGGCATC	pSC101-pPA construction by Gibson Assembly (GA) cloning
OL680	GGCTGACGCCGTTGGATACACCAAG	
OL1691	CTTGGTGTATCCAACGGCGTCAGCCAATTCGCGCGCGAAGGCGAA	
OL1692	GGTGTCAACGTCACTGCCCGCTTTCTCAGAATATTTGCCAGAACC	
OL713	GAAAGCGGGCAGTGACGTTGACACCCAAGGGAAAATTTATCAAAA	
OL1693	TATTTGATGCCTCTAGAGCTTGCATGCTTAATTTCTCCTCTTTAA	
OL2226	GGAACGACGCTAACCATCTTAATCATGCTTAATTTCTCCTCTT	pOrf20 construction by GA cloning
OL2227	CAGCTGGTTGAGAAGGCGAAATAACAAGCTCTAGAGGCATCAAA	
OL2228	ATTAAGATGGTTAGCGTCGTTCCG	
OL2229	TTATTTCGCCTTCTCAACCAGCTG	
OL1892	CATGCTTAATTTCTCCTCTT	pOrf36.1 construction by GA cloning
OL1893	CAAGCTCTAGAGGCATCAAA	
OL1894	AATTAAAGAGGAGAAATTAAGCATGAAATCAGCTGAAGACTG	
OL1895	TTTTATTTGATGCCTCTAGAGCTTGCTACCGGAAGAGTCCTTTCG	
OL1892	CATGCTTAATTTCTCCTCTT	pOrf38 construction by GA cloning
OL1893	CAAGCTCTAGAGGCATCAAA	
OL1900	AATTAAAGAGGAGAAATTAAGCATGAAAGATTTTAAGCTCAC	
OL1901	TTTTATTTGATGCCTCTAGAGCTTGTCATTTAAAACCTCTCTTAC	
OL1878	CTAGAGTCGACCTGCAGGCATGCAATGCAAGCTCTAGAGGCATC	pExcA construction by GA cloning
OL1879	GAGCTCGAATTCGCTAGCCCAAAAATTCTAGATGTGTGAAATT	
OL1636	TTTTTGGGCTAGCGAATTCGAGCTCTGGTATAGCGGGGGCGTGAT	
OL1637	TGCATGCCTGCAGGTCGACTCTAGATCAATACGTTCGTGGTAGCA	
OL1878	CTAGAGTCGACCTGCAGGCATGCAATGCAAGCTCTAGAGGCATC	pRepC construction by GA cloning
OL1879	GAGCTCGAATTCGCTAGCCCAAAAATTCTAGATGTGTGAAATT	
OL1638	TTTTTGGGCTAGCGAATTCGAGCTCCGTATGCTGTGGGAGGAAGA	
OL1639	TGCATGCCTGCAGGTCGACTCTAGATCACTCTTCAGAATCGTCAA	
OL2326	AAAGAAGCAGACCAAAAAAAAGTGAATGCCG	pOrf32 construction by GA cloning
OL2327	TCAGTAAAACTTCACGTTCGATTTCAGGG	
OL2328	CGGCATTCACTTTTTTTTGGTCTGCTTCTTTCATGCTTAATTTCTCCT CTT	
OL2329	GCCCTGAAATCGAACGTGAAGTTTTACTGACAAGCTCTAGAGGCAT CAAA	
OL374	ATGTCTAAAGGTGAAGAACTGTTC	

OL375	CACTGCCCGCTTTCCAGT	pR6K-P_{Tet}-sfGFP construction by GA cloning
OL871	CTGGAAAGCGGGCAGTGTAATTCCTAATTTTTGTTGACAC	
OL872	GTTCTTCACCTTTAGACATTTCACTTTTCTCTATCACTGA	
OL1616	TGATTTCTACGGTTTTTTTCTAAATAATGTGAGGTTCAAAGTGTAG GCTGGAGCTGCTTC	λred *parA* deletion
OL1617	GCACTTACGGTTATCAGTCATACTCAGCCCTTCATTGCCACATATG AATATCCTCCTTAG	
OL1620	TCGATCTGGTTCTGGGTATGGTGGCAATGAAGGGCTGAGTGTGTAG GCTGGAGCTGCTTC	λred *parB* deletion
OL1621	AACAGCACATATTTTTTCACGCAGCCCCCTTAATCATCATCATATG AATATCCTCCTTAG	
OL1622	CGTCCAGCATCTGTTCCCGGATGATGATTAAGGGGGCTGCGGTGTA GGCTGGAGCTGCTT	λred *nuc* deletion
OL1623	GTTAAAAATCAGGCGGCAGAGTTTCTGCCGCTGCTGTAGGCATATG AATATCCTCCTTAG	
OL2218	CATTTTTCGGTAGCGCCCCGACTGGCGAAGGAGTAAAACCGTGTA GGCTGGAGCTGCTTC	λred *orf19* deletion
OL2219	CATCTTAATCATTACCCTTCTCCCAGAAGTCTGAATTCAGCATATG AATATCCTCCTTAG	
OL2220	GTGAAAATTTTTCTGAATTCAGACTTCTGGGAGAAGGGTAGTGTAG GCTGGAGCTGCTTC	λred *orf20* deletion
OL2221	TGTGGTGATCATAAAGGGTCCTCTCTGGTCGCCGCGGGCCCATATG AATATCCTCCTTAG	
OL974	TCAGAAGAGGTCATAATGCACCTGTAAGGCTGTTTAGGAACTGGC ACGACAGGTTTCCCGA	Insertion P_{Tet}-*sfgfp-frt-kan-frt* in pOXA-48
OL975	CATTAGACGTATAATGTTTTTACTGATTCAAATCAGTACCCATATG AATATCCTCCTTAGT	
OL1721	TTTCAACGCGGCGGTGTTCCGCCGCGGTTAGTAAACGCATGTGTAG GCTGGAGCTGCTTC	λred *orf36.1* deletion
OL1722	AGACTGGCTGCATACTGTCAGACGTTTCATGAATGAAGATCATATG AATATCCTCCTTAG	
OL1825	AATACCGGAGTTCCCCCATGAAAGATTTTAAGCTCACCAGGTGTAG GCTGGAGCTGCTTC	λred *orf38*deletion
OL1826	CATCCTCAAAGAAAGAAATCATTTAAAACCTCTCTTACCCCATATG AATATCCTCCTTAG	
OL2284	AAAAAATGGGAGGATTAAGGGGGATTTCTCCCCCTCTTTTCCATAT GAATATCCTCCTTAG	λred *orf32* deletion
OL2285	AGTCTTTCGCGGTAGCGCCCCGACTGGCGAAGGAGTTGAAGTGTA GGCTGGAGCTGCTTC	
OL2286	CCATTTTTTACTGATAAACATCAAATATATGAGGTGTATTGTGTAG GCTGGAGCTGCTTC	λred *ssb* deletion
OL2287	TTGGCGGCCCGCGTCCTGCGGGCCGTTCTAACTCAAATACCCATAT GAATATCCTCCTTAG	
OL2288	AGAAAAAAATGCCCCTTTTCGAGGCAAATTTTTAACCGGTCCATAT GAATATCCTCCTTAG	λred *orf33* deletion
OL2289	CGGCCCGCAGGACGCGGGCCGCCAATCCAGAGGGGGGAGTGTGTA GGCTGGAGCTGCTTC	
OL1624	ACGGTTGATAAAGACAAAGATGGTATAGCGGGGGCGTGATGTGTA GGCTGGAGCTGCTTC	λred *excA* deletion
OL1625	CATGAAAAAACCGCCTTTTCAGGCGGTTTGGAAGTAAACACATAT GAATATCCTCCTTAG	
OL1626	TATACTCAGGTGGTTATAGTCGTATGCTGTGGGAGGAAGAGTGTAG GCTGGAGCTGCTTC	λred *repC* deletion
OL1627	AGCGGGGCAATACCTGCGAACCCGCTTTTACATAGTAAGACATAT GAATATCCTCCTTAG	
OL2209	CTCGAGCGCTCGGTACCCACAGAGAGCGTTTCAGGC	TAP_{kn}-Cas9-OXA48-pemI-agrB construction by insertion in SmaI-digested pBG52
OL2210	GCACTGAAGGATCCCCCCGTATACCCTATTCCCGGTGG	
OL602	GATCCTCTAGAGTCGACCTGCAGGC	TAP_{kn}-Cas9-OXA48-agrB construction by removing *pemI*, followed by phosphorylation and self-ligation
OL1131	GAATGACCTGTCACGGGATAATCCGGCGGCAAACAGCAAAGACGT	
OL2245	GTTTTGGCGGATGAGAGAAG	pBAD-DqlB construction by GA cloning
OL2246	ATGGAGAAACAGTAGAGAGTTGCG	
OL2247	CTCTCTACTGTTTCTCCATGTCAATCTGTTGACGTTCTTC	
OL2248	CTTCTCTCATCCGCCAAAACCGATGATCGAGATGTAACCC	
OL2566	AATTGTTTAGGCTCCCCCGCCTCCATCCGGGGCAA	TAP_{kn}-Cas9-OXA48-mut_agrB construction by quick change mutagenesis
OL2567	ATGGAGGCGGGGGAGCCTAAACAATTGACGGTTAA	
OL2696	GGAACATCTAATACGTTGACGTGAGGTAGCCGCTGTGA	pBAD-mut_dqlB construction by quick change mutagenesis
OL2697	CCTCACGTCAACGTATTAGATGTTCCCACTCCAGCCAG	

L1204-I1	CAAGCAGAAGACGGCATACGAGATCGTGATAGACCGGGGACTTAT CATCCAACCTGT	Tn-seq libraries
L1204-I2	CAAGCAGAAGACGGCATACGAGATGCCTAAAGACCGGGGACTTAT CATCCAACCTGT	

SI References

1. Yu, D. *et al.* An efficient recombination system for chromosome engineering in Escherichia coli. *Proc. Natl. Acad. Sci. U.S.A.* **97**, 5978–5983 (2000).
2. Royet, K., Parisot, N., Rodrigue, A., Gueguen, E. & Condemine, G. Identification by Tn-seq of Dickeya dadantii genes required for survival in chicory plants. *Mol Plant Pathol* **20**, 287–306 (2019).
3. Nolivos, S. *et al.* Role of AcrAB-TolC multidrug efflux pump in drug-resistance acquisition by plasmid transfer. *Science* **364**, 778–782 (2019).
4. Poirel, L., Bonnin, R. A. & Nordmann, P. Genetic Features of the Widespread Plasmid Coding for the Carbapenemase OXA-48. *Antimicrobial Agents and Chemotherapy* **56**, 559–562 (2012).
5. Reuter, A. *et al.* Targeted-antibacterial-plasmids (TAPs) combining conjugation and CRISPR/Cas systems achieve strain-specific antibacterial activity. *Nucleic Acids Res* **49**, 3584–3598 (2021).
6. Goodman, A. L. *et al.* Identifying genetic determinants needed to establish a human gut symbiont in its habitat. *Cell Host Microbe* **6**, 279–289 (2009).
7. Datsenko, K. A. & Wanner, B. L. One-step inactivation of chromosomal genes in Escherichia coli K-12 using PCR products. *Proc. Natl. Acad. Sci. U.S.A.* **97**, 6640–6645 (2000).
8. Choi, K.-H. & Schweizer, H. P. mini-Tn7 insertion in bacteria with single attTn7 sites: example Pseudomonas aeruginosa. *Nat Protoc* **1**, 153–161 (2006).
9. Zaslaver, A. *et al.* A comprehensive library of fluorescent transcriptional reporters for Escherichia coli. *Nature Methods* **3**, 623–628 (2006).
10. Fraikin, N., Couturier, A. & Lesterlin, C. A palette of bright and photostable monomeric fluorescent proteins for bacterial time-lapse imaging. Preprint at https://doi.org/10.1101/2024.03.28.587235 (2024).
