# Supplementary material for: Genetic determinants of pOXA-48 plasmid maintenance and propagation in Escherichia coli
Source: Nat Commun. 2025 Aug 19;16:7734. doi: 10.1038/s41467-025-62404-7 (PMC12365149; doi:10.1038/s41467-025-62404-7)
Supplement: Supplementary file 2 — Description of Additional Supplementary Files [file 41467_2025_62404_MOESM2_ESM.pdf]

Title: Supplementary Movie 1

Description: Transconjugant viability following acquisition of the orf20 mutant plasmid under selective pressure. Donor and recipient cells were mixed for 4 h on a filter before being imaged every 10 min by time-lapse microscopy, with a continuous flow of fresh medium supplemented with ampicillin. Recipient cells, exhibiting red mCherry fluorescence, are sensitive to ampicillin. In the absence of complementation of the orf20 mutation by the pOrf20 plasmid (R without pOrf20, right panel), some recipient cells appear elongated with an increased width, continuing to elongate over time before undergoing bulging and eventual cell lysis. Non-fluorescent donor cells and transconjugants (which exhibit both mCherry and sfGFP fluorescence) are resistant to ampicillin due to expression of the blaOXA-48 gene encoded by pOXA-48. Donors E. coli ilvA::PBiofab-tetR / pOXA-48- Δorf20-PtetR-sfGFP/ pOrf20 (LY4161); recipients left panel: E. coli ilvA::Ptac□mcherry / pOrf20 (LY4140), right panel E. coli ilvA::Ptac-mcherry (LY1593). Scale bar: 10 µm

Title: Supplementary Data 1

Description: Tn-seq data analysis
